# Mechanisms Underlying Unconscious Processing and Their Alterations in Post-traumatic Stress Disorder: Neuroimaging of Zero Monetary Outcomes Contextually Framed as “No Losses” vs. “No Gains”

**DOI:** 10.3389/fnins.2020.604867

**Published:** 2020-12-16

**Authors:** Igor Elman, Jaymin Upadhyay, Steven Lowen, Keerthana Karunakaran, Mark Albanese, David Borsook

**Affiliations:** ^1^Center for Pain and the Brain, Department of Anesthesiology, Boston Children’s Hospital, Harvard Medical School, Boston, MA, United States; ^2^Cambridge Health Alliance, Harvard Medical School, Cambridge, MA, United States; ^3^Department of Psychiatry, McLean Hospital, Harvard Medical School, Belmont, MA, United States; ^4^Insulet Corporation, Acton, MA, United States

**Keywords:** fMRI, PTSD, striatum, prediction error, prefrontal cortex, insula, nucleus accumbens

## Abstract

Although unconscious processing is a key element of mental operation, its neural correlates have not been established. Also, clinical observations suggest that unconscious processing may be involved in the pathophysiology of post-traumatic stress disorder (PTSD), but the neurobiological mechanisms underlying such impairments remain unknown. The purpose of the present study was to examine putative mechanisms underlying unconscious processing by healthy participants and to determine whether these mechanisms may be altered in PTSD patients. Twenty patients with PTSD and 27 healthy individuals were administered a validated wheel of fortune-type gambling task during functional magnetic resonance imaging (fMRI). Unconscious processing was elicited using unconscious contextual framing of the zero monetary outcomes as “no loss,” “no gain” or as “neutral.” Brief passive visual processing of the “no loss” vs. “no gain” contrast by healthy participants yielded bilateral frontal-, temporal- and insular cortices and striatal activations. Between-group comparison revealed smaller activity in the left anterior prefrontal-, left dorsolateral prefrontal-, right temporal- and right insular cortices and in bilateral striatum in PTSD patients with the left dorsolateral prefrontal cortex activity been more pronounced in those with greater PTSD severity. These observations implicate frontal-, temporal-, and insular cortices along with the striatum in the putative mechanisms underlying unconscious processing of the monetary outcomes. Additionally, our results support the hypothesis that PTSD is associated with primary cortical and subcortical alterations involved in the above processes and that these alterations may be related to some aspects of PTSD symptomatology.

## Introduction

Arising outside of conscious awareness (Dijksterhuis, 2004), unconscious processing constitutes a key element of mental function (Bargh, 2019) that has evolved beyond immediate survival to support an instantaneous and effortless responsivity to internal and external stimuli (Greenwald et al., 1996). Unconscious processing does not exist in isolation, but is rather integrated within broader cognitive-, emotional-, and motivational operations (De Houwer, 2019), each of which exhibits a unique role ascertaining an optimal adjustability to complex internal and environmental challenges (Kahneman et al., 2011; Greenwald and Banaji, 2017). Deliberate logical and quantitative thinking, informed by cost/benefit considerations and aimed at profit maximization, could override unconscious processing (Darlow and Sloman, 2010) to yield a rational yet at times rigid and unspontaneous pattern of decisions and social interactions (Dijksterhuis, 2004; Newlin and Weinstein, 2015). Failure of the rational override conversely contributes to cognitive/perceptual biases (Greenwald and Banaji, 1995) as well as to impulsive and potentially disadvantageous decisions and behavioral choices (Dijksterhuis, 2004; Koob and Volkow, 2016). In order to improve outcomes in social- and clinical arenas it is important to better understand unconscious processing and how it is implemented in the human brain (Keren and Schul, 2009).

From the clinical perspective, unconscious processing has been implicated in the course of various mood-, anxiety-, thought-, and stressor-related disorders. Post-traumatic stress disorder (PTSD) may be construed as a prototype of such disorders as it amalgamates the above elements (American Psychiatric Association, 2013). There are several lines of evidence that link altered unconscious processing and PTSD. In addition to PTSD’s association with unconscious threat-related physiologic and behavioral responses manifested in anxiety and in conditioned fear (Elman and Borsook, 2018), there are clinical and even diagnostic features of PTSD itself that point to unconscious biases. The most notable of this is the implicit memories phenomenon (McNally, 1997; Southwick et al., 1999) contained within the PTSD “core component” (Bryant, 2019) of the re-experiencing i.e., the “B” diagnostic criteria (American Psychiatric Association, 2013). Automatic “negative alterations in cognitions” is another key PTSD component (Hassin et al., 2006) encoded in the “D” diagnostic criteria e.g., negative thoughts and assumptions (American Psychiatric Association, 2013). Consequently, without the presence of at least one of the unconscious biases, the diagnosis of PTSD is nearly impossible to make. Moreover, unconscious trauma reminders (VanElzakker et al., 2014; Musazzi et al., 2018), irrational (Hyland et al., 2014) and impulsive (Kotler et al., 2001; Roley et al., 2017) decision making may precipitate illness’ exacerbations (American Psychiatric Association, 2013) while psychotherapeutic approaches e.g., Eye Movement Desensitization and Reprocessing (Landin-Romero et al., 2018) and pharmacologic GABAergic agents (Jovasevic et al., 2015) that affect unconscious processing (VanElzakker et al., 2014) hold therapeutic promise for PTSD patients. However, unconscious processing’s neural correlates in PTSD patients are unknown.

Hitherto, unconscious processing has been addressed in psychological literature focused on judgments and decisions e.g., cognitive shortcuts and heuristic biases (Evans and Stanovich, 2013), habitual valuation system (Rangel et al., 2008), probability weighting (Fennell and Baddeley, 2012), perceptual illusions (Chen et al., 2018) or prejudicial attitudes and stereotyping (Kurdi et al., 2019). As to neurobiological factors, *predictive coding* is an unconscious inference (Friston et al., 2014) resulting from the sensory inputs that are modulated by contextually linked internal representations derived from the interpretation of prior experiences formed in the hierarchically higher cortical centers (Brown and Brune, 2012; Kiefer, 2012). Unconscious cognition is ostensibly inaccessible to self-reports and the inquiry into the underlying mechanisms is limited, in part, by a paucity of laboratory-based procedures that induce strong and reproducible activation of the major unconscious processing systems that can be controlled with respect to the “amount” of the administered stimulus (Brooks and Stein, 2014).

Money, an easily quantifiable and ubiquitously recognized stimulus that can be incorporated into unconscious probabilistic judgment and decision-making tasks (Knutson and Cooper, 2005), provides a meaningful framework for human neurobiological research by integrating concepts from experimental psychology, economics and computational neuroscience. According to the *Prospect Theory* (Kahneman and Tversky, 1979), a distinct and unconscious cognition is brought to bear in evaluating the prospects of a monetary offer and in anticipating and assessing the eventual outcomes, namely the editing of a prospect as a gain or a loss with respect to a neutral point. An approach we (Hopper et al., 2008; Elman et al., 2009) have developed to examine unconscious processing in humans is the measurements of functional magnetic resonance imaging (fMRI) signal changes evoked by wheel-of-fortune-like spinners. Owing to the passive nature, visual processing of the spinners is devoid of decisions-weighing, of behaviors aimed at reward-seeking or avoidance of punishment (Alcaro and Panksepp, 2011) as well as of an active choice prompting counterfactual comparison between the obtained and alternative outcomes (Alcaro and Panksepp, 2011). The unpromising (i.e., bad)-, promising (i.e., good)- or intermediate (i.e., close to affective neutrality) spinners establish controlled states of unconscious framing (De Martino et al., 2006) in which the neutral point falls somewhere between the extreme monetary values on each display (Breiter et al., 2001; Hopper et al., 2008). By usurping cognitive resources (Betsch et al., 2001), keeping pace with the brevity (i.e., 5.5 s) of the monetary outcomes’ presentation hinders conscious tracking and updating the product of the ongoing trial and of the overall task (Fazio et al., 1986; Breiter et al., 2001) to allow the isolation of unconscious processing (Cassotti et al., 2012) associated with losses or gains, framed by the expected outcomes. The resultant configuration is of particular interest given that changes in the activity of dopamine neurons reflect deviations of outcomes from expectations (Schultz, 2017) namely, prediction error (Delgado et al., 2008; Niv and Schoenbaum, 2008; Solms, 2017). Zero outcome displayed on each spinner with 33.33% probability evokes, akin to the “half full” vs. “half empty” prediction error minimization (Hohwy, 2012) reflected in either relief (“no loss” for the bad spinner) or disappointment (“no gain” for the good spinner) implicit judgments and attitudes (Mellers, 2000; Kim et al., 2006) without conscious awareness of their causality (Dijksterhuis, 2004). Therefore, a significant prediction errors’ difference on the bad zero (BZ) vs. good zero (GZ) fMRI signals’ contrast is indicative of the brain susceptibility to the framing variation effect (De Martino et al., 2006), that is to say, an unconscious interpretation that could have been rejected by a conscious effort afforded via a longer duration of the stimulus exposure (Betsch et al., 2001; Schultz, 2007) recognizing the identity of the zero outcomes heralding no change in the material assets.

The aims of the present study was to examine how the BZ vs. GZ monetary outcomes impact upon brain functioning in healthy- and PTSD participants using the same validated monetary task as on our prior studies (Hopper et al., 2008; Elman et al., 2009). We hypothesized the involvement of the striatum and other terminal dopamine fields commonly connected to automatic computation of conditional probabilities (Trepel et al., 2005; Rangel et al., 2008; Elman and Borsook, 2016). Because unconscious processing in general, and contextual framing in particular have not yet been methodically addressed in PTSD literature and theoretical considerations on this score are not unambiguous, directional prediction on PTSD patients’ responses (greater or smaller effect on the BZ vs. GZ monetary contrast) was not sufficiently justified (Elman et al., 2018) and the hypothesis was formulated in terms of PTSD-related alterations. The value of using our monetary procedure is that it allows a conclusive interpretation of the findings. Control level fMRI signal changes in PTSD patients would suggest intact brain function in connection with this sort of monetary stimuli. In contrast, decrements on the above stimuli measurements would suggest a psychopathological basis of unconscious processing in PTSD patients. Yet again, heightened responses to either BZ or GZ outcome would be consistent with sensitized state of the respective stimulus; the latter could be suggestive of hypervigilance (American Psychiatric Association, 2013) toward aversive stimuli typical of PTSD symptomatology (Bryant, 2019). Increased brain responses to one stimulus associated with proportional signal decrements during another stimulus would support the notion that “no loss” and “no gain” responsivity are inversely related phenomena (Elman et al., 2018).

## Materials and Methods

### Participants

All procedures were carried out in accordance with the Declaration of Helsinki, and the protocol was approved by the Institutional Review Boards of McLean Hospital. Twenty patients diagnosed with PTSD as determined by the Structured Clinical Interview for DSM-IV-TR Axis I Disorders (First et al., 2002) and Clinician-Administered PTSD Scale (CAPS) (Weathers et al., 2001) and 27 healthy individuals were included in this study after the procedures were fully explained and written informed consent was given. Eight PTSD patients and six healthy controls were excluded due to motion or missing button box responses to identify the spinners (see below). All study participants reported no physical illnesses; their good physical health and right-handedness were determined by the respective Cornell Medical Index Health Questionnaire (Seymour, 1976) and Edinburgh Handedness Inventory (Oldfield, 1971). Participants’ recent drug and alcohol consumption was ruled out by negative results on urine toxicology screen and breathalyzer test. Participants with a history of schizophrenic-, paranoid-, other psychotic-, bipolar-, non-PTSD anxiety-or substance dependence disorder were excluded. Given the high rate of depressive comorbidity in PTSD (O’Donnell et al., 2004), patients with onset of major depressive disorder after the traumatic event that precipitated PTSD were allowed to participate. We excluded the use within the previous month of any potentially confounding medications or drugs (e.g., opioids, psychostimulants, cannabinoids, dopaminergic or antidopaminergic agents e.g., antipsychotics, and mood stabilizers and antidepressants with prominent catecholaminergic effects such as tricyclics, bupropion, mirtazapine, venlafaxine, and duloxetine). Gains vs. losses fMRI contrasts from these participants are reported elsewhere (Elman et al., 2009).

### Protocol

**FIGURE 1:**
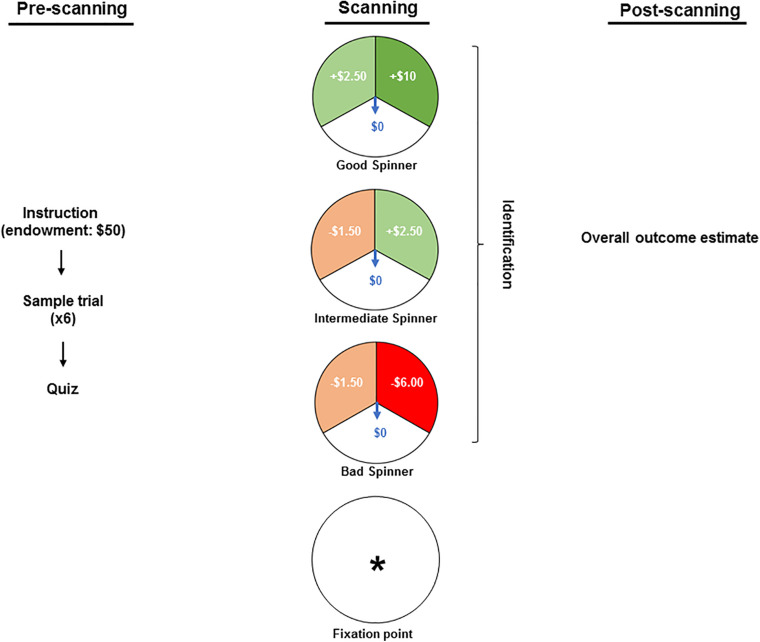
The pre-scanning stage included reading the instruction text wherein participants were informed about an endowment of $50 granted for participation in a game and about a possibility of losing some or all of this stake, retaining or increasing it. Thereafter, the participants observed a sample set of six trials and each participant’s familiarity with the spinners’ types was confirmed via a computerized quiz. In the scanner, the stimuli were interspersed by a fixation point (an asterisk) and comprised of three different spinners: a “bad” spinner that generates a large loss ($6.00), a small loss ($1.50) or no loss ($0.00); a “good” spinner that generates a large gain ($10.00), a small gain ($2.50) or no gain ($0.00); and an “intermediate” spinner that generates a small loss ($1.50), a small gain ($2.50) or neither a loss nor a gain ($0.00). While in the scanner, participants were requested to press one of the three buttons to identify the projected spinner as a measure of attention. At the conclusion of the scanning (i.e., post-scanning stage), participants’ estimates of their overall monetary gain or loss were obtained to provide a measure of attention and understanding.

Figure 1 summarizes the protocol. By reading the instruction text, participants were informed about an endowment of $50 granted for participation in a game of chance wherein they might lose some or all of this stake, retain or increase it. Thereafter all questions were answered, and participants observed a sample set of six trials with all spinners’ types. Each participant’s familiarity with the spinners’ types and their outcomes was then confirmed via a brief computerized quiz, completion of which was conditioned on the correct understanding of the task. The stimuli were interspersed by a fixation point (an asterisk) and comprised of three different spinners: a “bad” spinner that generated a large loss ($6.00), a small loss ($1.50) or no loss ($0.00); a “good” spinner that generated a large gain ($10.00), a small gain ($2.50) or no gain ($0.00); and an “intermediate” spinner that generated a small loss ($1.50), a small gain ($2.50) or neither a loss nor a gain ($0.00). Gains were set larger than losses to adjust for the greater salience attributed to the magnitude of monetary outcomes used in the present study (Redelmeier et al., 1993; Harinck et al., 2007). The contextual framing commenced with viewing one of the three spinners, which remained static for the first 0.5 s and was then overlaid by a rotating arrow for 5.5 s. As a measure of attention, during that phase, participants were requested to press one of the three buttons to identify the projected spinner. The outcome phase was marked by the rotating arrow’s halting in one of the spinners’ three sectors. The sector in which it halted then flashed for 5.5 s to highlight the outcome. The outcome phase was concluded with 0.5 s of blank screen. Unbeknownst to the participants, the trial sequence was pseudorandom and identical for all participants so that each spinner and outcome were both preceded and followed equally often by all spinner × outcome combinations yielding a final net winning of $78.50. At the conclusion of the scanning participants’ estimates of their overall monetary gain or loss were obtained to provide a measure of attention and understanding.

### fMRI Data Acquisition, Processing and Analyses

Functional magnetic resonance imaging data were acquired on a 3-Tesla Siemens Trio MR Imaging System (Siemens AG, Erlangen, Germany) using a gradient echo, echo planar imaging (EPI) sequence, with repetition time/echo time = 2000/30 msec, 220 mm × 220 mm field of view (FOV), 3-mm coronal slices starting from the anterior pole, no gap, right-left readout, 64 × 64 pixel, full k-space acquisition, no sensitivity encoding [SENSE] acceleration; pulse sequence-enhanced version of the Siemens epibold. The scanning session was subdivided into nine blocks of 19 trials each, separated by 2–4-min rest periods; total fMRI acquisition time was 9 × 4:04 min. Automatic second order shimming was performed over the fMRI imaging volume before acquisition. After the functional scans, participants had a conventional T1 scan performed on the same fMRI volume with identical slice prescription (matched warped; FOV = 220 mm × 220 mm and 3-mm coronal slices acquired covering the whole brain). A standard T1-weighted magnetization prepared rapid gradient echo (MPRAGE) three-dimensional (FOV = 256 mm × 256 mm × 170 mm, 256 × 256 × 128) was also collected. Low resolution T1-weighted image were then aligned to the high resolution T1-weighted MPRAGE images with twelve degrees of freedom, using fMRIB’s Linear Image Registration Tool (FLIRT). We employ a high-resolution, T1-weighted “match-warped” EPI image that increases the precision of the alignment between the fMRI dataset and the high-resolution MPRAGE (Frederick et al., 2007). The MPRAGE was then aligned to Montreal Neurological Institute (MNI) 152 standard brain with FLIRT using twelve degrees of freedom. Rendering of the fMRI results in MNI space was performed after concatenating the three alignments into a single matrix. A summary of this registration was monitored for each run of each participant.

### fMRI Data Processing and Statistical Analyses

Each trial block was checked for missing button box responses, excessive head motion during fMRI acquisition and image registration (see below), in that order. If fewer than two trials passed quality control, the participant was dropped from the subsequent data analysis. Runs were deemed valid if participants pressed any spinner identification button at least once; if no buttons were pressed, the run was discarded.

Functional magnetic resonance imaging data were processed with FSL 6.0 (FMRIB Analysis Group, 2020). Preprocessing procedures included the following steps: (1) An in-house despiking filter was applied to all functional data sets; (2) All images within a scan were aligned to image #60 (in the middle), using MCFLIRT (Jenkinson et al., 2002), with six degrees of freedom. If the maximum deviation from this reference exceeded 3.0 mm (the smaller voxel dimension) the scan was discarded; (3) Slice timing correction was performed; (4) Non-brain voxels were removed; (5) Spatial filtering was performed, using a Gaussian kernel with 5 mm full width half maximum; (6) Global normalization was performed, such that the average over all voxels and images was fixed at 10^4^ and (7) Temporal filtering was performed, using a non-linear high pass filter with a cutoff of 18 s. Regularized autocorrelation functions were independently estimated for each voxel, using temporal Tukey prewhitening (Woolrich et al., 2001). General linear model (GLM) regressors comprised of (1) “On-period” representing the $0 outcome on each of the three spinners types, set to unity during that outcome, and zero otherwise; (2) One for each of the six motion estimates obtained from motion correction. Each non-motion regressor was subjected to a linear filter modeling the hemodynamic response function, having a gamma impulse response, width of 3 s, and mean lag of 6 s. Non-motion regressors were further subjected to the same temporal filter that was applied to the data. Regressors varied in a counterbalanced manner across runs, but each run was identical over all participants. The results of this analysis were discarded except for the residuals. A principal component analysis was performed on the 5000 voxels in the residuals that had the largest variance. The first eight components were retained and used as additional nuisance regressors (without temporal filtering) in a new generalized linear model, using the same pre-processed functional data. All regressors were retained for later spectral analysis. From the resulting parameter estimates, the outcomes’ contrasts were calculated.

Mechanisms underlying unconscious processing effects were examined by quantifying the fMRI response to the BZ, GZ and intermediate zero (IZ) conditions. Within-subject contrasts were subsequently generated for (1) BZ vs. GZ, (2) BZ vs. IZ, and (3) GZ vs. IZ conditions. In group-level, mixed-effects (FLAME 1) analyses, *t*-test results for each voxel were converted to Z scores and thresholded to *p* < 0.01, at first uncorrected for multiple comparisons. All voxels with less significant activations (or deactivations of any magnitude) were excluded from further study. Remaining voxels were then collected into continuous clusters. Using Gaussian random field theory, a significance level was associated with each cluster, enabling a correction for multiple comparisons across the whole brain. Clusters with corrected significance were thresholded at *p* < 0.05. All group-level activation and deactivation maps were superimposed on the MNI-152 brain template (1 mm^3^).

## Results

### Participants

Patients with PTSD were not significantly different from healthy controls with respect to age (mean ± SD = 33.0 ± 10.5 vs. 28.4 ± 8.3 years; *t*_1_,_45_ = 1.70, *p* = 0.10), race (white/non-white = 16/4 vs. 22/5; Fisher’s exact test *p* = 1.0), gender distribution (male/female = 12/8 vs. 12/15; Fisher’s exact test *p* = 0.38), education status (with-/without high school degree 20/0 vs. 26/1, Fisher’s exact test *p* = 0.71) or estimates of the net monetary gains across the experiment ($54.6 ± 38.6 vs. $54.5 ± 63.9 years; *t*_1_,_44_ = 0.005, *p* = 1.0). As planned, the respective CAPS- (70.8 ± 17.9) and Beck Depression Inventory-II (BDI-II) (18.4 ± 13.5) scores (Wang and Gorenstein, 2013) were significantly higher (*p* < 0.01) in PTSD- than in healthy participants. Psychotropic medications taken by the PTSD patients included selective serotonin reuptake inhibitors (*n* = 6), trazodone, (*n* = 2), buspirone (*n* = 2), gabapentin (*n* = 1), topiramate (*n* = 1), and clonazepam (*n* = 1).

### Voxelwise BZ vs. GZ Contrast

**FIGURE 2:**
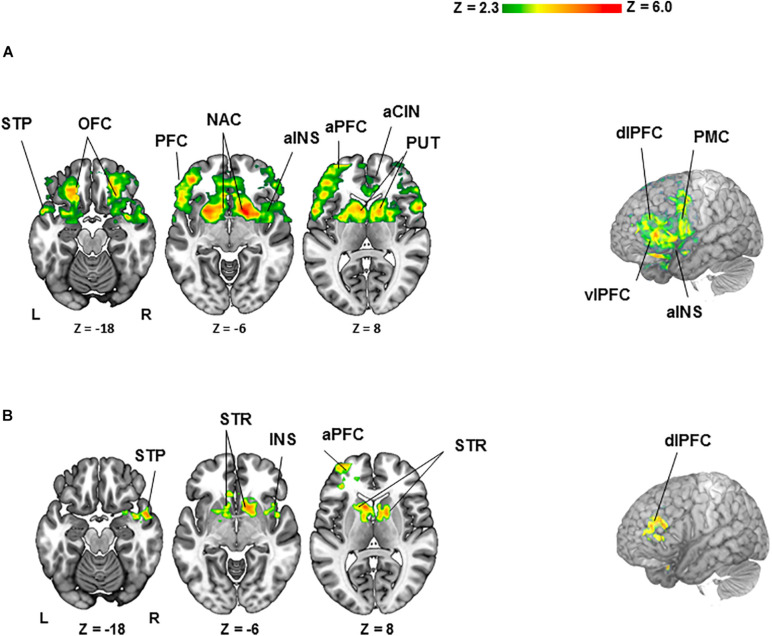
Clusters of activation (colored) obtained from voxelwise contrasts of the bad- minus good spinner zero (BZ-GZ) projected onto a background (grayscale) representing participants’ mean high-resolution anatomic image (*z* > 2.3, with a cluster-defining threshold of *p* = 0.05 and corrected for multiple comparisons). Coordinates are in accordance with the Montreal Neurological Institute (MNI) space. **(A)** For healthy participants (*n* = 27), significant activation was detected in frontal-, temporal-, and insular cortices and striatal regions. **(B)** For healthy (*n* = 27) > PTSD (*n* = 20) contrast, significant activation was detected in frontal-, temporal-, and insular cortices and striatal regions. Terms of location: a, anterior; dl, dorsolateral; vl, ventrolateral. Cortical: CIN, cingulate; INS, insula; OFC, orbitofrontal cortex; PFC, prefrontal cortex; PMC, premotor cortex; STP, superior temporal lobe. Subcortical: NAC, nucleus accumbens; PUT, putamen; STR, striatum (caudate, pallidum and putamen).

For the BZ vs. GZ contrast (Figure 2A and Table 1), healthy participants demonstrated robust BOLD activation in the cortical- (i.e., anterior cingulate; anterior insula; anterior-, dorsolateral-, orbitofrontal-, and ventrolateral regions of the prefrontal cortex; premotor cortex and superior temporal lobe) and subcortical regions (caudate, nucleus accumbens, pallidum, and putamen). Conversely, PTSD patients did not show any significant activation or deactivation throughout the brain for the BZ vs. GZ contrast. Group comparison (healthy- vs. PTSD participants) revealed greater activation across the left anterior prefrontal-, left dorsolateral prefrontal (DLPC)-, right temporal-, and right insular cortices along with the bilateral caudate, nucleus accumbens, pallidum and putamen (Figure 2B and Table 1).

**TABLE 1:** BZ vs. GZ Activations in Healthy Participants.

	*X*	*Y*	*Z*	Z-statistic (max value)
Nucleus Accumbens (L)	−10	14	−6	4.64
Nucleus Accumbens (R)	12	11	−6	4.84
Caudate (L)	−7	12	8	4.71
Caudate (R)	14	12	4	5.03
Putamen (L)	−23	5	0	5.05
Putamen (R)	22	6	−2	6.06
Pallidum (L)	−14	−2	−3	4.00
Pallidum (R)	20	4	−4	5.25
Superior Frontal Gyrus (L)	−14	8	63	4.60
Superior Frontal Gyrus (R)	20	26	60	4.47
Frontal Pole (L)	−46	42	2	5.29
Inferior Frontal Gyrus (R)	58	13	5	5.05
Orbital Frontal (L)	−20	31	−23	5.05
Orbital Frontal (R)	27	30	−21	5.28
Precentral Gyrus (L)	−47	5	43	4.61
Precentral Gyrus (R)	53	5	32	4.56
Anterior Cingulate (R)	5	5	32	4.32
Paracingulate (L)	−3	40	−10	4.45
Anterior Insula (L)	−35	22	−7	3.02
Anterior Insula (R)	42	12	−8	3.46
Superior Temporal Pole (L)	−55	10	−18	4.22
Superior Temporal Pole (R)	50	3	−15	4.01
**BZ vs. GZ Activations in Healthy- vs. PTSD Participants**				

	***X***	***Y***	***Z***	**Z-statistic (max value)**

Nucleus Accumbens (L)	−9	14	−6	2.85
Nucleus Accumbens (R)	12	15	−5	3.57
Caudate (L)	−9	13	8	3.71
Caudate (R)	14	9	8	3.34
Putamen (R)	24	5	−2	2.87
Pallidum (L)	−11	1	2	4.42
Pallidum (R)	15	0	3	3.34
Frontal Pole (L)	−48	42	16	4.05
Insula (R)	41	12	−7	3.00
Superior Temporal Pole (R)	50	7	−15	4.05
**BZ vs. IZ Activations in Healthy Participants**				

	***X***	***Y***	***Z***	**Z-statistic (max value)**

Nucleus Accumbens (L)	−10	15	−8	3.32
Nucleus Accumbens (R)	9	18	−2	2.85
Caudate (L)	−13	6	11	3.13
Caudate (R)	11	14	0	3.23
Putamen (L)	−21	4	−4	3.09
Putamen (R)	30	−2	7	3.59
Pallidum (L)	−19	−2	−1	3.56
Pallidum (R)	15	6	1	3.72
Frontal Pole (L)	−36	47	−3	4.50
Frontal Pole (R)	30	37	11	3.02
Paracingulate (R)	13	43	20	4.00
Anterior Cingulate	0	32	10	3.30
Orbital Frontal (L)	−23	24	−22	4.19
Orbital Frontal (R)	28	31	−17	3.17
Anterior Insula (L)	−28	25	−3	3.19
Superior Temporal Pole (L)	−54	13	−20	3.06
Superior Temporal Pole (R)	53	18	−17	3.56
**BZ vs. IZ Activations in Healthy- vs. PTSD Participants**				

	***X***	***Y***	***Z***	**Z-statistic (max value)**

Frontal Pole (L)	−36	45	−3	4.00
**GZ vs. IZ Deactivations in Healthy participants**				

	***X***	***Y***	***Z***	**Z-statistic (max value)**

Caudate (L)	−14	14	12	3.01
Caudate (R)	17	15	12	3.07
Putamen (L)	−27	6	−5	3.27
Putamen (R)	18	7	−7	4.37
Superior Frontal Gyrus (L)	−12	−15	61	4.36
Middle Frontal Gyrus (L)	−34	11	51	3.49
Frontal Pole (L)	−3	63	17	3.55
Inferior Frontal Gyrus (L)	−52	27	−4	3.79
Anterior Insula (L)	−33	4	12	3.54

*Post hoc* analyses assessed potential confounds stemming from depression or medication for the BZ vs. GZ contrast. A comparison of responses between PTSD patients with (*n* = 16) or without (*n* = 4) major depression did not reveal significant differences (FDR corrected *p*-value = 0.85). In line with this observation, regression analyses incorporating individual patient BDI-II scores into the GLM model did not show a significant effect for the BZ vs. GZ contrasts. Furthermore, a comparison among PTSD patients on (*n* = 12) vs. off (*n* = 8) medications also did not yield significant differences (FDR corrected *p*-value = 0.19).

**FIGURE 3:**
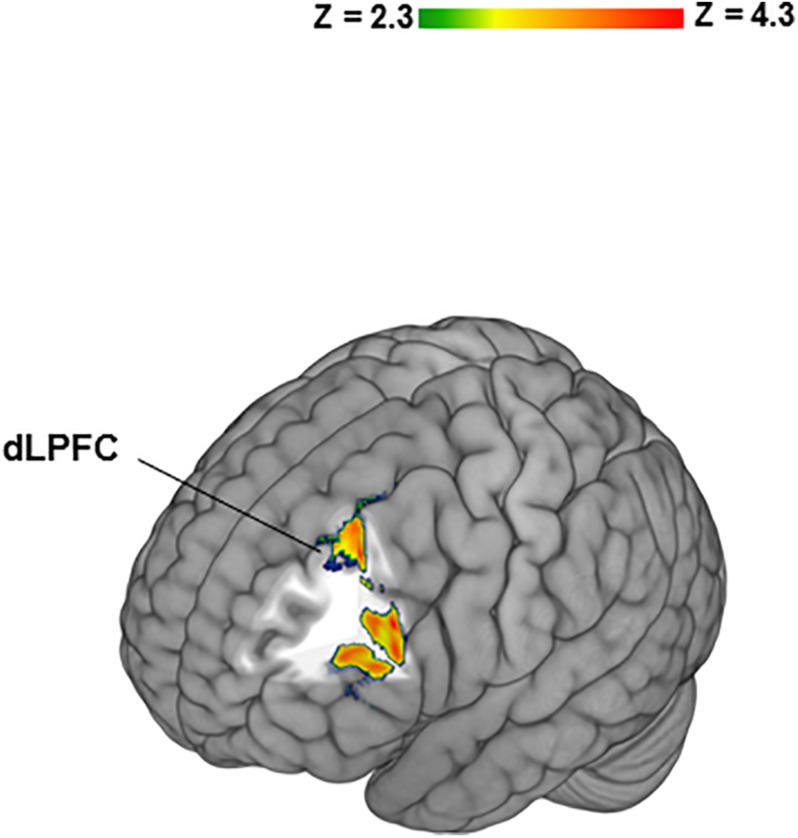
Significant relationship between Clinician-Administered PTSD Scale (CAPS) scores and left dorsolateral prefrontal cortex (DLPFC) activity in PTSD patients. Individual CAPS scores were incorporated as regressors of interest in higher level General linear model analyses on the BZ vs. GZ contrast.

To determine potential contributions of “no losses” and “no gains” separately to the differences between BZ and GZ, each was contrasted to IZ. In healthy participants the BZ vs. IZ contrast yielded significant activation in bilateral frontal, temporal, insular cortices along with the dorsal and ventral striata (Table 1). PTSD patients did not show any significant fMRI activation or deactivation for the BZ vs. IZ contrast. Group-level differences (i.e., healthy- vs. PTSD participants) for the BZ vs. IZ contrast was evident in increased activation for the left frontal pole (Table 1). In healthy volunteers, the GZ vs. IZ contrast led to significant deactivation in the bilateral frontal cortex, caudate and putamen (Table 1). PTSD patients presented no changes on that contrast. No significant group differences were overserved for the GZ vs. IZ contrast. When individual CAPS scores were incorporated as regressors of interest in higher level GLM analyses on the BZ vs. GZ contrast an association was observed between CAPS scores and left DLPFC activity (Figure 3).

## Discussion

### Unconscious Processing by Healthy Brain

According to Lem (1965), “*Everyone knows that dragons don’t exist*…*. They were all, one might say, non-existent, but each non-existed in an entirely different way*.” Here a contrast between non-existent monetary losses vs. gains in healthy participants yielded significant bilateral activations within the extensive salience and threat response circuitry, critical for the survival of individuals and species namely, frontal-, temporal-, insular- and striatal regions (Elman and Borsook, 2018). The functional and structural segregation of the “no loss” and “no gain” signals provides neuroanatomical credence to the Prospect Theory (Kahneman and Tversky, 1979) attributing to framing rather than to plain changes in the asset position emphasized by the Expected Utility Theory (Mellers, 2000), a key role in the evaluation of outcomes. The validity of the activations in the bilateral prefrontal and cingulate cortices and dorsal and ventral striata is strengthen since they are consistent with the meta-analyses of 35 studies implicating these brain regions in the processing of prediction error (Garrison et al., 2013). Our present work thus extends the prior findings by suggesting that these regions’ role is not specific to the instrumental and Pavlovian conditioning, but actually generalizes to passive forms of contextual framing. Insular activations on the BZ vs. GZ and vs. IZ contrasts supports its general prediction function (Siman-Tov et al., 2019) and not just that specific to the aversive domain (Garrison et al., 2013).

In a previous fMRI framing study (De Martino et al., 2006), participants performed a financial decision-making task selecting hypothetical trials (not real money) framed as gains or losses (e.g., keeping £20 out of £50 or losing £30 out of £50) that were displayed as numbers and pie probability charts (De Martino et al., 2006). The framing effect, operationalized via consistent preference of one out of the two equivalent options, was associated with bilateral amygdala activity (De Martino et al., 2006; Roiser et al., 2009). An active choice component (Hart and Izquierdo, 2017) and conscious cognitions arising in the context an alternative outcome (Nicolle et al., 2011) may explain the divergent amygdala findings on that (De Martino et al., 2006) and the present study where there was no amygdala activation. Other brain regions may be also engaged as patients with bilateral amygdala degeneration (Talmi et al., 2010) displayed intact framing capacity on the same task (De Martino et al., 2006). Another main difference between the two tasks is the revealed outcome or feedback (c.f., Sheynin et al., 2013) that triggers “what could have happened” types of contextual processing with the consequent emotional responses that may be blunted in PTSD patients (Elman et al., 2005; Hopper et al., 2008). So, the task employed here does not allow us to firmly conclude that emotional numbing to both negative and positive outcome values is not implicated in the lack of significant activation to the BZ vs. IZ and GZ vs. IZ contrasts in the PTSD group. Disentangling this component would require an exclusively visual-processing task that did not employ the value function.

Valence plays a key role in neuronal responses to the outcome-predicting events. Specifically, neuronal activity is increased by events with better values than predicted, is uninfluenced by events that are as good as predicted, and is reduced by events that are worse than predicted (Schultz et al., 1997; Spoormaker et al., 2011). The results of separate contrasts of BZ and GZ with IZ accordingly indicated significantly higher responsivity to “no loss”- and significantly lower responsivity to “no gain” outcomes. Even though, as per the Prospect Theory (Kahneman and Tversky, 1979), the gains were chosen to be larger than the losses in order to adjust for the heightened salience attributed to the former than to the latter, “no loss” (expected value: $2.50), as compared to “no gain” (expected value: $4.17), engaged a more extensive salience neurocircuitry with emotional and cognitive constituents merging on the brain networks comprising the medial prefrontal cortex, the insula and the nucleus accumbens. Such generalization of the heightened loss salience attributed to the opposite to loss omission state (Erdeniz and Done, 2019) may be adaptive from a phylogenetic perspective as it improves coping mechanisms by reinforcing risk aversive choices and behaviors. More research is warranted to determine which characteristics of loss omission e.g., passive vs. active (Kim et al., 2006) or the magnitude of the stakes (Yechiam and Hochman, 2014) are mostly salient and whether PTSD and disease states that are linked to other types of losses such as depressive- or anxiety disorders (Keyes et al., 2014) have common salience-related pathophysiologies.

### Unconscious Processing in PTSD

Post-traumatic stress disorder patients showed an altered framing and consequent prediction error processing evident in the lack of respective brain activations and deactivations to both, “no losses” and “no gains” stimuli. This pattern suggesting that PTSD patients, in addition to being unmoved by life’s near misses, might also be indifferent to life’s misfires has relevance in the clinical domains outside of behavioral finance. Differences in the framing function may determine whether an individual retains control of the symptoms or whether the symptoms attain control over the individual. The editing operation frames a prospect of continued illness or a successful recovery with respect to an endurable amount of disturbing symptoms to allow reformulation of the coping strategies in accordance with the amount of psychological suffering that a patient is capable of accepting, thus altering the reference (neutral) point (Elman and Borsook, 2019). All other things being the same, symptoms up to the limit of this new neutral state may not be perceived as an aversive experience. Future studies employing a variety of contextual framing tasks are needed to continue addressing this entity. This is important because the monetary stimulus employed on the present study is qualitatively different from the types of stimuli that have been implicated in exacerbations of the PTSD disease process (Moser et al., 2015; Steenkamp et al., 2017). It is possible that different stimuli have unique effects on the unconscious processing regulation including framing and prediction error.

Contextual processing is an essential component of psychosocial wellbeing, mediated in part via the μ-opioid system (Chen et al., 2020). Given that a partial opioid agonist, buprenorphine, has been successfully tried in PTSD patients (Seal et al., 2016; Lake et al., 2019) it would be of interest to test whether buprenorphine is able to normalize the abovementioned blunted responses. It has been proposed that some psychopathological conditions with excessive endogenous opioid function e.g., PTSD (Liberzon and Abelson, 2016; Elman and Borsook, 2019), schizophrenia (Elman et al., 2006) or addiction (Elman and Borsook, 2019) are associated with aberrant contextual processing (Waters et al., 2006; Anderson and Hanslmayr, 2014). Examination of neurocircuitries for these categories of mental illnesses reveals overlapping differences between patients and control groups in cortical and subcortical limbic structures (Elman and Borsook, 2018) implicated in re-experiencing and recall type of symptoms e.g., hallucinations- or craving-related memories (Waters et al., 2006; Elman and Borsook, 2016). Such findings, however, do not yet conclusively demonstrate unitary nosology because PTSD entails manifestations of altered neurocircuitry in other domains be it reward function or motivational regulation (Elman and Borsook, 2019). An approach reflected in the Research Domain Criteria (Silveira et al., 2020) is to divide that kind of multidimensional constructs into domains based on the underlying circuitry or system. Each domain can be then studied separately, which in and of itself may be a daunting task given the complexity of the systems involved. For instance, the re-experiencing construct encompasses overlapping threat- (acute, potential, or sustained) vs. memory (declarative or working) vs. attention- vs. perception- and cognitive control elements pertaining to the respective aspects of the intrusive traumatic memories (RDoC Domains Constructs, 2020). And so, before specific clinical correlates of potentially altered unconscious processing in PTSD are to be investigated, it is first necessary to show that such alteration exists. The latter, and not the former, was the objective of the present project. Its methods may be applied to PTSD populations in the future studies in order to address the question of neurocircuitry subserving the re-experience and recall effects.

Our data accord with a substantial body of PTSD literature documenting disruptions of the cortico-striatal-limbic meshwork underlying emotional, motivational and cognitive functions (Britton et al., 2006; Elman et al., 2018; Duval et al., 2020). Prevailing PTSD theories likewise ascribe the hippocampus an important role in the illness’ pathophysiology (Joshi et al., 2020) particularly as it pertains to conscious experiences (Behrendt, 2017) including contextual processing-derived (Liberzon and Abelson, 2016) associative learning (Lambert and McLaughlin, 2019). This may be why the present design is unable to inform the question as to whether conscious aspects of contextual processing deficits by the PTSD patients are implicated in hippocampal function (Joshi et al., 2020). There is a need in continued focus on this important area and its role in the PTSD pathophysiology.

The study participants were right-handed, which is determined by the dominance of the left hemisphere consistent with the correlation between PTSD symptomatology and left DLPFC activations. Prior connectome PTSD work revealed reduced connectivity between the left lateral prefrontal regions and the regions implicated in the processing of salience (Misaki et al., 2018) viz., caudate (Peters et al., 2016), pallidum (Ahrens et al., 2018) and putamen (Ulrich et al., 2014) striatal nuclei. It is plausible that regulatory deficits in the subordination of the subcortical circuits mediating unconscious content to the higher order cortical centers (Elman and Borsook, 2018) contributed to the lack of the BZ vs. GZ brain responses in PTSD patients. PTSD therapeutic armamentarium may thus be enriched through targeting the aforesaid top-down and bottom-up “two-system” corticostriatal construct (Becerra et al., 2001; LeDoux and Pine, 2016; Elman and Borsook, 2018).

### Caveats

In this study we produced a 33.33% expectancy condition for the zero outcome by making it one of the three possible outcomes on each of the spinners. We did not incorporate the 0 and 100% zero expectancy conditions, though, which would have constituted a respectively maximal- and neutral prediction error states. A limitation of the cross-sectional design that could require prospective and/or twin studies is its inability to resolve the risk factor versus acquired origin (Pitman et al., 2006) of the contextual framing hyporesponsivity in PTSD patients. Chronic stress associated with the persistent reliving of the traumatic event adversely affects one of its primary target organ, the brain (Yehuda and LeDoux, 2007), with local (Sekiguchi et al., 2013) and diffuse tissue reductions (Kasai et al., 2008; Clausen et al., 2020) as well as changes in cerebral perfusion and metabolism (Gold et al., 2011; MacNamara et al., 2016). In light of this, it is tempting to conjecture that the origin of the unconscious processing dysfunction found in this study is stress damage to the brain. However, it is also possible that a preexisting genetic or acquired risk factor, which is manifest in contextual framing is imparting vulnerability for PTSD. How might the latter possibility come to pass? In clinical work, particularly with veterans, it is common to hear that the most thrilling event of their lives was successfully escaping from an ambush echoing the commonly accepted psychological notion that active avoidance of punishment is reinforcing. This may not be the case for predisposed people, which could diminish their avoidance of stressful situations with heightened potential for trauma exposure to the point of helplessness, which is an essential precursor for the development of PTSD (Pivovarova et al., 2016). This causality is far from been settled, however, with consistently documented counterfactual thoughts (Erwin et al., 2018; Kelley et al., 2019) and ruminations (Blix et al., 2018; Scott et al., 2018) regarding the index trauma as features of PTSD. In short, further studies are necessary to pursue unconscious processing of active choice outcomes that are linked vs. not linked to prior traumatic experiences. Moreover, to enhance neuropsychopathology characterization and formulation of treatment plans, clinical PTSD assessments might benefit from specific questioning concerning emotional and motivational significance of both, distinctively valenced trauma-related content and more ambiguous contextually framed perceptions not necessarily relevant to the index trauma.

Sensory perception is, however, not a mere duplicate of the objective reality. Rather, it is a set of images and impressions that are actively generated by the brain e.g., a misperception of the zero outcome as a negative or positive event that seems to be muted in PTSD patients. Explicitly, whereas an acute stress normatively amplifies an unconscious and holistic (Curby and Moerel, 2019) perception heavily relying on intuitive and habitual elements (Yu, 2016), our findings suggest that chronic stress in the form of PTSD on the contrary favors more rational processing. On a prior study (Hopper et al., 2008), PTSD patients correctly rated lower expectancy from the bad vs. the good spinner. In contrast to healthy controls they, however, failed to display extra satisfaction that is normally incumbent upon omission of the negative outcome, which may be another instance of a rational processing. In fact, an etiologically and pathophysiologically linked (Smoller, 2016; Mgoqi-Mbalo et al., 2017) construct of major depression is likewise associated with a lack of misperception of at least some of reality aspects typical of mental health (Moore and Fresco, 2012). Even so, the fast and intuitive unconscious processing is underlying the lion’s share of the healthy thinking apparatus (Kahneman, 2011) and its diminution/impairment in neuropsychiatric disorders such as PTSD and major depression requires continued consideration by researchers and clinicians alike.

Strengthening some of the default mode networks (DMN) induced by trauma and by the ensuing PTSD (Suo et al., 2019) may render the brain more attuned to past memories, to introspection and to other types of internal experiences (Kernbach et al., 2018) with corresponding reduction in the responsivity to externally directed tasks (Laurienti, 2004) limiting the engagement by the emotions and motivations ordinarily arising in the context of a game of chance. DLPFC activity during the bad- vs. good zero contrast were more pronounced in those with greater PTSD severity. If chronic stress associated with PTSD (Elman and Borsook, 2019) increases the DMN nerve traffic (Zhu et al., 2020) then we might indeed expect a relative increase in the left DLPFC activity against the backdrop of the overall diminished activity aimed at prioritization of personally relevant content at the expense of external demands (Turnbull et al., 2019) placed by the monetary task. Disorders with stress-related attenuation of cerebral blood flow and metabolism, such as PTSD, major depression or schizophrenia (Bogdanov and Schwabe, 2016; Park et al., 2016) do show relative increases in the left DLPFC activity consistent with its inefficient functioning (Manoach et al., 1999; MacNamara et al., 2016). Therefore, abnormalities of the DMN and/or the dorsal fronto-parietal attention network, entrusted with goal-directed attentional performance (Kryger et al., 2017) may both contribute to PTSD pathophysiology.

A number of additional limitations apply to this fMRI study were partially addressed here and have previously been discussed in greater detail (Elman et al., 2009, 2018). On the whole, these limitations include the cross-sectional design, the correlational nature of some results and the ecological validity of the employed stimulus. Further considerations that should be noted pertain to the scientific value of the unconscious processing construct, the nature of contextual framing, the medication status and the use of the DSM-IV-TR PTSD criteria (American Psychiatric Association, 2000). Behaviorists are focused on behavioral responses to external stimuli and outright omit unconscious processing as an unamendable to direct measurement intervening variable linking the two former entities (Miles, 1966). Cognitive scientists by and large emphasize conscious mental processes including the ones that are automatically triggered by external stimuli (Hunt, 1985; Bargh, 2019). To generate new leads for understanding unconscious processing and to harness these insights into improved preventive, diagnostic and therapeutic strategies (Bargh, 2019), clinicians and scientists are striving to merge modern behavioral and cognitive theories with earlier psychoanalytic perspectives. Thus, while we recognize that others may disagree with some of the unconscious processing theoretical formulations (Bargh, 2019), our task builds upon the major psychological theories to accord with the modern definition of unconscious processing (Bargh, 2019) in that it embroils automatic (cognitive psychology) generation of internal mental representations that are triggered by external stimuli (behaviorism) albeit in the absence of a conscious awareness or/and an intent (psychoanalytic theory).

Exigencies of patient recruitment did not allow the exclusion of all factors that could have confounded the study results. There is a considerable amount of data supporting the involvement of serotonergic mechanisms in the generation of prediction errors (Greenberg et al., 2019; Sebold et al., 2019) that could have been affected by the patients’ medications. Even though the diminished contextual framing effect in the PTSD group remained significant after the adjustment for the current medication use, the question regarding the precise role of PTSD pharmacotherapy in modulation of unconscious processing still remains open and more inquiry controlling for medications status is needed. Furthermore, the presence of major depression may alter the brain’s contextual framing system (Greenberg et al., 2019). Given the substantial comorbidity of PTSD with major depression (O’Donnell et al., 2004), however, implementing this as an exclusion factor would have ruled out a high percentage of patient candidates as to make recruitment unfeasible. Even if adequate patients were recruited with the above constraints, the resultant group would have likely be unrepresentative of the universe of PTSD patients. However, we attempted to balance the recruiting efforts with pragmatics by excluding so called “endogenous” depression.

There are noteworthy changes from DSM-IV TR to DSM 5 PTSD diagnostic criteria including, but not limited to the expansion of the type of incorporated symptomatology and to the deletion of a seemingly “indispensable” (Pivovarova et al., 2016) “fear, helplessness or horror” trauma response criterion (Pai et al., 2017). Consequently, in comparison to DSM-5, DSM-IV TR PTSD criteria yield a greater diagnostic sensitivity viz., fewer false negatives’ rates (Schaal et al., 2015; Schnyder et al., 2015). This raises the possibility of a difference in the present study outcomes using the newer nosology and underscores the importance of the ongoing quest for more refined diagnostic tools particularly in the areas of the exposure to- and the trauma *per se* definitions (North et al., 2016). Identification of reliable neuroimaging PTSD biomarkers thus represents a timely scientific endeavor that could be translated into effective diagnostic and therapeutic procedures (Fenster et al., 2018).

## Conclusion

Our observations support the thesis that an extended set of brain regions including frontal-, temporal-, and insular cortices along with the striatum may be involved in the mediation of unconscious processing inherent in contextual framing. The congruence of our findings with the results from other monetary stimuli research (Coricelli et al., 2005; Kim et al., 2006; Johnson et al., 2019) lend further credence to the suggestion of a “single mind” operation switching the conscious and unconscious modes as needed for arriving to optimal decisions and choices while engaging overlapping brain areas (Kim et al., 2006). That the observed effects are involved in PTSD pathophysiology is supported by a significant correlation between PTSD severity and DLPFC activations. Therefore, unconscious processing alterations remain a credible target of both, pharmacological and non-pharmacological interventions, the efficacy of which could be monitored via symptomatic improvement and its correlation with the fMRI findings. Our results call for further research aimed at understanding the distinctive features of adaptive- vis-à-vis detrimental effects of chronic stress and their potential role in PTSD’s unique pathophysiology. Further dissection of the unconscious processing subsystem could yield valuable data toward understanding of motivated behavior and its aberration in PTSD and perhaps in other neuropsychiatric disorders with partially overlapping neurobiology such as depressive- and anxiety syndromes, addictions and schizophrenia.

## Data Availability Statement

The raw data supporting the conclusions of this article will be made available by the authors, without undue reservation.

## Ethics Statement

The studies involving human participants were reviewed and approved by the Institutional Review Board of McLean Hospital. The patients/participants provided their written informed consent to participate in this study.

## Author Contributions

IE, SL, and DB conceived the study and designed the experiments. SL coded the imaging algorithms, set up imaging parameters, programmed the study task for the performance in the magnet, and performed the primary analyses of the imaging data. IE and DB oversaw and performed the experiments. JU provided secondary analyses of the data. MA contributed to the clinical expertise in interpretation of clinical and neuroimaging data. SL, JU, KK, and IE drafted the Materials and Methods and Results sections. IE drafted the manuscript. All authors contributed to interpretation of data and reviewed the submitted version of the manuscript.

## Conflict of Interest

SL was employed by the company Insulet Corporation. The remaining authors declare that the research was conducted in the absence of any commercial or financial relationships that could be construed as a potential conflict of interest.
